# Frequency of Metabolic Syndrome in Chronic Hepatitis C Patients: Findings From a Lower Middle Income Country

**DOI:** 10.7759/cureus.11975

**Published:** 2020-12-08

**Authors:** Saeeda Fouzia Qasim, Ajmaal Jami, Paras Imran, Romana Mushtaque, Rashid Naseem Khan

**Affiliations:** 1 Internal Medicine, Liaquat College of Medicine and Dentistry, Karachi, PAK; 2 Endocrinology, Diabetes and Metabolism, Jinnah Postgraduate Medical Centre, Karachi, PAK; 3 Medicine, Hamdard College of Medicine and Dentistry, Hamdard University, Karachi, PAK; 4 Medicine, Civil Hospital Karachi, Karachi, PAK; 5 Internal Medicine, Kulsoom Bai Valika Social Security Site Hospital, Karachi, PAK

**Keywords:** metabolic syndrome, hcv, dyslipidemia, obesity, hepatocellular carcinoma, diabetes mellitus

## Abstract

Introduction

The world over, hepatitis C virus (HCV) engenders the risk of developing chronic hepatitis, cirrhosis and hepatocellular carcinoma (HCC). It has many extrahepatic manifestations, among which diabetes and metabolic syndrome (MetS) has been increasingly recognized and has become an active research field. The current study aimed to ascertain the frequency of MetS in chronic hepatitis C patients and to curb its long-term adverse outcomes.

Methods

In our cross-sectional analysis, a total of 331 subjects diagnosed with chronic HCV were registered from June 2017 to November 2018 in two tertiary care hospitals of Karachi, Pakistan. Metabolic syndrome (MetS) was delineated following the National Cholesterol Education Program (NCEP) Adult Treatment Panel III (ATP III). Categorical variables were compared by using the Chi-square test, and a significant P value was at the value of < 0.05.

Results

We found that adults of 40 - 49 years of age were the worst sufferers of hepatitis C. Out of the total 331 patients of hepatitis C, 97 (29.3%) cases were suffering from metabolic syndrome.

Conclusion

Prevalence of MetS is substantial among HCV-infected individuals Therefore estimation of MetS in individuals with HCV infection is imperative and patients should be educated for lifestyle modification, diet, and weight control. However, we cannot generalized the results of our study as it was done in some tertiary care centres, so additional surveys are warranted to know the actual prevalence of MetS in our entire population.

## Introduction

Ubiquitously hepatitis C virus (HCV) is a crucial public health problem and can give rise to hepatic and extra-hepatic complications. According to the World Health Organization (WHO) census 2013, over 71 million people across the globe are suffering from HCV [1]. In Pakistan, the prevalence of HCV is approximately 6% [2]. The National Hepatitis Study documented that eight million individuals are suffering from this life-threatening virus and among them, many individuals are unaware of having this viral disease, resulting in delayed disease detection and therapy. Karachi is one of the biggest cities in Pakistan and has an occurrence rate of 5.5% [3].

In chronic hepatitis C (CHC) sufferers, MetS is a common association. According to studies, HCV has no personal linkage with the lipid metabolism in hepatocytes, however it interrupts the circulating lipoprotein metabolism. HCV virus affects very low-density lipoprotein (VLDL) particles and takes over the formative pathway of the VLDL particle for production of an infectious particle named lipoprotein viral particle (LVP) [4], which circulates in the bloodstream and actively exchanges HCV among lipoproteins and hence contributes to the pathophysiology of the disease. Hepatitis C causes insulin resistance, which affects the hormone-sensitive lipase, leading to more conversion of triglyceride into free fatty acid. Damage of the liver due to hepatitis C enables the triglyceride metabolisms, hence the free fatty acid increase in the blood causing hypertriglyceridemia [5-7].

MetS is a cluster of problems that increases the risk of developing various diseases. It is related to the probability of subsequent development of insulin resistance, type 2 diabetes mellitus, and micro and macrovascular complications [8]. Microalbuminuria and hyperuricemia are also observed to be part of this cluster [9]. The presence of these disorders hastens the possibility of developing hepatocellular carcinoma (HCC) [10]. In a systematic review, Dyal et al. concluded that CHC patients with diabetes are at greater likelihood for HCC development [11]. Hepatitis C has a relationship with insulin resistance and type 2 diabetes mellitus. Findings from a previous studies by Negro et al. [12] indicate that resistance to antiviral treatment may be related to insulin resistance.

The delayed diagnosis of hepatitis C eventually results in cirrhosis, HCC, and cardiovascular complications related to MetS [9,13]. A systematic review and meta-analysis by Ambrosino et al. showed a significantly increased menace of cardio-cerebrovascular diseases in HCV patients [14]. Several studies also had been done in other parts of the world to find an association between MetS and CHC. Our study intended to ascertain the frequency of MetS in victims of CHC.

## Materials and methods

A total of 331 individuals with confirmed HCV infection were enrolled in our cross-sectional analysis. The sample size was calculated by Open Epi version 3 (Open Source Epidemiologic Statistics for Public Health; www.OpenEpi.com) assuming a 95% confidence level, frequency of MetS 26%, and 10% non-response rate. Patients who visited the liver clinic in the department of medicine in two tertiary care hospitals of Karachi, Pakistan from June 2017 to November 2018 with positive hepatitis C by polymerase chain reaction (PCR) were selected. All adult male and female patients with age >18 with the diagnosis of hepatitis C were included and all patients were treatment-naive. Patients with a history of alcohol consumption, decompensate chronic liver disease, pregnant females, concomitant malignancy, heart and renal disease, co-infection with hepatitis B and HIV/AIDS were excluded from the study.

MetS were delineated based on the National Cholesterol Education Program (NCEP) Adult Treatment Panel III (ATP III) standards. Patients with three or more of the following five criteria were labelled as MetS [15]: waist circumference ≥ 80 centimeters (cms) in females and ≥ 90 cms in males, blood pressure ≥130/85 mmHg or higher, fasting blood sugar (FBS) ≥ 100 mg /dl, fasting high-density lipoprotein (HDL) cholesterol ≤40 mg/ dl in men or ≤ 50 mg/dl in women, fasting triglycerides (TG) level ≥150 mg/dl [16].

Anthropometric data including waist and hip circumference, height, and weight were recorded by the trained staff, according to standardized criteria recommended by WHO [17]. Flexible inch tape was used for the estimation of the waist and hip circumference. The waist was measured at the midpoint between the costal margin and the iliac crest while the hip circumference was measured at the widest part of the buttocks. Measurements of height and weight were done by using stadiometers and calibrated digital weighing scales respectively. Body mass index (BMI) was computed by putting the values in the standardized formula of weight in Kg/height in meters square. Blood pressure was measured by a digital automatic blood pressure device (Omron HEM - 907 model; OMRON, Kyoto, Japan), in a seated position. Blood samples were drawn after 10 hours of fasting. All blood investigations were done by the Clinical Diagnostic Laboratories from the same hospitals.

The diagnosis of HCV was established based on a positive serum antibodies test and its authentication was done by qualitative HCV ribonucleic acid (RNA). The HCV antibodies were checked by enzyme-linked immunosorbent assay (ELISA) method using Biotek ELx 800 (BioTek Instruments, Winooski, Vermont, USA). Qualitative HCV RNA was conducted by Abbott Real-Time PCR Assay m2000sp and m2000rt (Abbott Laboratories, Abbott Park, Illinois, USA).

Lipid profile, fasting blood glucose, uric acid, alanine aminotransferase (ALT) and gamma-glutamyl transferase (GGT) were done using Architect ci4100 analyzer by Abbott Core Laboratory. The data entry and analysis were done by using the Statistical Package for Social Sciences (SPSS) version 19 (IBM Corp., Armonk, NY, USA). Categorical variables (such as gender, course of hepatitis C) were expressed in frequency(%) while numerical variables were expressed as mean ± SD of BMI, waist circumference, lipid profile, and fasting blood glucose.

The categorical variables were compared by using chi-square and p-value will be significant as <0.05. Ethical permission was given from the Institutional Review Board (IRB) of Liaquat College of Medicine and Dentistry (IRB-28/LCMD/11/2019) and written informed consent was signed by all participants.

## Results

More than half (57%) of the patients belonged to 40 - 49 years of age, followed by 21% in 50 - 59 years and 11% in the age group between 30 - 39 years. The average age of patients was 47.5 +4.85 and 122 (36.9%) were males and 209 (63.1%) were females. As high as 252 (76%) of the patients were hypertensive and 126 (38%) were diabetic. MetS was found in 97 (29.3%) of the patients out of the total of 331 subjects. Among a total of 97 MetS patients, systolic blood pressure >130 mmHg was found in 67 (69.1%) patients while diastolic >85 mmHg in 46 (47.4%) patients. Triglycerides above 150 mg/dl were found in 55 (56.70%) patients and increase total cholesterol in 47 (48.45%) patients. High-density lipoprotein was lower than <40 mg/dl in 15 (4.5%) patients only. Seventy-one (73.2%) patients with MetS had high fasting plasma glucose. We observed that uric acid level in our patients was in the range between 3.0 mg/dl to 8.30 mg/dl. No statistically significant (p>0.05) difference was observed between the frequency of metabolic syndrome and the duration of hepatitis C (Tables 1, 2).

**Table 1:** Demographic and Clinical features of the Study Participants (n = 331)

Variable	n(%) or mean + SD
Age (years)	47.5±4.85
Male	122(36.9)
Female	209(63.1)
Systolic Blood Pressure(mmHg)	128 ±10.9
Diastolic Blood Pressure (mmHg)	75.8 ±7.3
Waist Circumference(cms)	80.2 ± 11.0
BMI(Kg/m^2^)	29.3± 5.4
Fasting Blood Sugar(mg/dl)	111 ± 5.8
Triglycerides	144.4 ± 4.4
Total Cholesterol	124 ± 4.6
High-Density Lipoprotein	38.4 + 1.36
Uric Acid	5.6 ± 2.0
Aspart Aminotransferase	68 ± 3.6
Gamma Glutamyl Transferase	128± 4.1
Duration of Hepatitis C (years)	
Less than 1 year	43(13)
1 - 3 years	97(29.3)
More than 3 years	191(57.7)

**Table 2:** Biochemical Parameters of Study Patients with or without MS

	Metabolic Syndrome (n=97)		p-value (95% CI)
Biochemical Parameters	Yes	No	
Systolic Blood Pressure(mmHg) > 130 mmHg	67(69.1)	30(30.9)	0.0049 (58.91% -78.09%)
Diastolic Blood Pressure (mmHg) >85 mmHg	46(47.4)	51(52.6)	0.1377 (37.17% - 57.80%)
Fasting Blood Sugar(mg/dl) >110mg/dl	71(73.2)	26(26.8)	0.0451 (63.25% - 81.69%)
Triglycerides >150mg/dl	55(56.7)	42(43.3)	0.0026 (46.25% - 66.73%)
High Density Lipoprotein <40mg/dl(men) <50mg/dl (women)	15(15.5)	82(84.5)	< 0.0001 (8.95% - 24.26%)
Waist circumference >102 cm (>40 in) Men/>88 cm (>35 in) Women	35(36.1)	62(63.9)	< 0.0001 (26.59% - 46.48%)

Ten (3%) of the individuals suffering from hepatitis C for less than one year were also the patients of MetS and 27 (8%) of the subjects with hepatitis C for one to three years were also the patients of this syndrome. A total of 60 (18%) of the sufferers of hepatitis C for more than three years were also patients of MetS (Table 3).

**Table 3:** Frequency of MetS with Duration of Hepatitis C Infection (n=331)

	Metabolic Syndrome			p-value
Duration of Hepatitis C (years)	Yes	No	Total	
Less than 1 year	10(10.3)	33(89.7)	43	0.5298
1 - 3 years	27(27.8)	70(62.2)	97	
More than 3 years	60(61.9)	131(38.1)	191	
Total	97	234	331	

## Discussion

Hepatitis C is highly prevalent in Pakistan and is linked with increased morbidity and mortality. We found that adults of 40 - 49 years of age are the worst sufferers of hepatitis C. There were 97 (29.3%) cases suffering from MetS out of the total sampled patients (331) of hepatitis C. A study from Pakistan by Bashir et al. showed a prevalence of MetS to be 17.78 %, quite lower than in our study [18]. This significant difference between the two studies from Pakistan is probably because of the difference in ethnicity. The study by Bashir et al. was conducted at Rahim Yar Khan, the Punjab side of Pakistan so checked in the Punjabi population while our study was conducted in the metropolitan city of Karachi, with people from multiple ethnicities including Punjabi, Sindhi, Pakhtoon, Urdu speaking, and Baluchi. Another reason for the difference is probably the increased number of female subjects (63%) in our study in contrast to 33% in the study by Bashir et al. Oliveira et al. reported 21.6% MetS in Hepatitis C virus-infected patients while a study from Taiwan showed a prevalence of 25.6% in CHC patients [19,20]. On comparing our study with another study done by Hung et al., with results showing 24.7% MetS in CHC patients, however, they had more patients with genotype 1 [21]. From the previous studies done across Pakistan, we already know that genotype 3 is predominant in our hepatitis C patients [22].

As regards the lipid profile, only 15 had significantly lower HDL-C. Fifty-five participants had higher levels of cholesterol, triglycerides (TGs) and low-density lipoprotein cholesterol (LDL-C). These findings are juxtaposed to the Taiwan study that imparted significantly lower cholesterol, LDL-C levels, HDL-C and TG levels in anti-HCV positive individuals [21]. In our study of 97 patients with MetS, 85% were overweight and 88% had a high waist circumference. The same results were demonstrated in the study done by Hung et al. [21]. Moucari et al. did a study on 600 patients with chronic hepatitis and found that 32% of the patients associated with MetS had insulin-resistant diabetes [23].

Hypertension, overweight, central obesity, insulin resistance, and type 2 diabetes mellitus have terribly confederated with MetS. Consequently, it is crucial to keep track of insulin resistance, weight, and blood pressure, as it would be instrumental to save our patients from adverse outcomes of this syndrome. Especially it is paramount to maintain healthy body weight as working on this risk factor solely can decrease the occurrence of co-morbidities and perhaps impedes the succession of chronic liver disease. Early diagnosis of hepatitis C is very much important as a timely intervention with new direct-acting antivirals (DAA) which can significantly decrease the risk associated with metabolic manifestations of chronic HCV [24].

## Conclusions

There is a high preponderance of MetS in chronic HCV infected individuals. Estimation of MetS in HCV-infected individuals may conceivably be requisite and patients should be educated for lifestyle modification, diet, and weight control. The limitation of our study is that it was conducted in some tertiary care hospitals, therefore the results cannot be generalized to the entire population. Further extensive analyses are imperative to reckon the prevalence of MetS in our hepatitis C positive population. Another limitation is that this study was conducted in the metropolitan city of Karachi on subjects with multiple ethnicities, so more studies are required in each ethnic group. It is also paramount to appraise whether the response to antiviral treatments can be improved with interventions involving remodelling of the lifestyle. 
